# MEGARes and AMR++, v3.0: an updated comprehensive database of antimicrobial resistance determinants and an improved software pipeline for classification using high-throughput sequencing

**DOI:** 10.1093/nar/gkac1047

**Published:** 2022-11-16

**Authors:** Nathalie Bonin, Enrique Doster, Hannah Worley, Lee J Pinnell, Jonathan E Bravo, Peter Ferm, Simone Marini, Mattia Prosperi, Noelle Noyes, Paul S Morley, Christina Boucher

**Affiliations:** Department of Computer and Information Science and Engineering, University of Florida, Gainesville, FL, USA; VERO Program, Veterinary Medicine and Biomedical Sciences, Texas A&M University, Canyon, TX, USA; Food-Centric Corridor, Infectious Disease Laboratory, Department of Veterinary Population Medicine, College of Veterinary Medicine, University of Minnesota, St. Paul, MN, USA; VERO Program, Veterinary Medicine and Biomedical Sciences, Texas A&M University, Canyon, TX, USA; Department of Computer and Information Science and Engineering, University of Florida, Gainesville, FL, USA; Food-Centric Corridor, Infectious Disease Laboratory, Department of Veterinary Population Medicine, College of Veterinary Medicine, University of Minnesota, St. Paul, MN, USA; Data Intelligence Systems Lab, Department of Epidemiology, College of Public Health and Health Professions and College of Medicine, University of Florida, Gainesville, FL, USA; Data Intelligence Systems Lab, Department of Epidemiology, College of Public Health and Health Professions and College of Medicine, University of Florida, Gainesville, FL, USA; Food-Centric Corridor, Infectious Disease Laboratory, Department of Veterinary Population Medicine, College of Veterinary Medicine, University of Minnesota, St. Paul, MN, USA; VERO Program, Veterinary Medicine and Biomedical Sciences, Texas A&M University, Canyon, TX, USA; Department of Computer and Information Science and Engineering, University of Florida, Gainesville, FL, USA

## Abstract

Antimicrobial resistance (AMR) is considered a critical threat to public health, and genomic/metagenomic investigations featuring high-throughput analysis of sequence data are increasingly common and important. We previously introduced MEGARes, a comprehensive AMR database with an acyclic hierarchical annotation structure that facilitates high-throughput computational analysis, as well as AMR++, a customized bioinformatic pipeline specifically designed to use MEGARes in high-throughput analysis for characterizing AMR genes (ARGs) in metagenomic sequence data. Here, we present MEGARes v3.0, a comprehensive database of published ARG sequences for antimicrobial drugs, biocides, and metals, and AMR++ v3.0, an update to our customized bioinformatic pipeline for high-throughput analysis of metagenomic data (available at MEGLab.org). Database annotations have been expanded to include information regarding specific genomic locations for single-nucleotide polymorphisms (SNPs) and insertions and/or deletions (indels) when required by specific ARGs for resistance expression, and the updated AMR++ pipeline uses this information to check for presence of resistance-conferring genetic variants in metagenomic sequenced reads. This new information encompasses 337 ARGs, whose resistance-conferring variants could not previously be confirmed in such a manner. In MEGARes 3.0, the nodes of the acyclic hierarchical ontology include 4 antimicrobial compound types, 59 resistance classes, 233 mechanisms and 1448 gene groups that classify the 8733 accessions.

## INTRODUCTION

Antimicrobial drugs, biocides and metals often impair or kill microbes. However, some microbes can resist the deleterious effects of these compounds via antimicrobial resistance (AMR) mechanisms. These diverse AMR mechanisms allow microbes to persist despite the presence of such compounds, and thus threaten the efficacy of protocols that rely on antimicrobial drugs, biocides or metals. Such protocols include therapeutic antibiotic regimens (such as those used in clinical human and veterinary medicine), as well as sanitation procedures and antimicrobial properties of manufactured products.

The mechanisms that confer AMR are diverse but are typically driven by antimicrobial resistance genes (ARGs) that confer antimicrobial effects when expressed within living bacterial cells. Therefore, molecular approaches have become integral to AMR research, and the use of genomic sequencing to identify ARGs in individual bacteria or entire microbial communities is fundamental to research and surveillance efforts. We previously introduced the MEGARes (v1.0) database of gene sequences for ARGs and the AMR++ (v1.0) bioinformatic pipeline to facilitate analysis of large-scale datasets, such as those produced from metagenomic sequencing (1). We note that AMR++ is designed to take as input a set of metagenomic sequence reads, which are then aligned to the MEGARes database via short read alignment; the output of the read alignment is used to predict the diversity and abundance of ARGs. MEGARes represented a unique contribution to ARG databases by implementing an acyclical hierarchical classification ontology that facilitates high throughput bioinformatic processing and statistical analysis. This structure allows for binning of alignment counts into mutually exclusive categories, which can then be aggregated to each of the complete levels of the hierarchical ontology. This prevents multiple counting of alignments at different taxonomic levels and thus accommodates downstream statistical analyses. We subsequently introduced the significantly expanded MEGARes v2.0, which expanded upon MEGARes v1.0 to include ARGs that target biocides and metals, in addition to antimicrobial drugs (2). Since publication, MEGARes v1.0 has been cited in more than 250 manuscripts, and MEGARes v2.0 has been cited in >120 papers. These citations relate to a variety of applications, such as research targeting AMR in aquatic environments (3,4), agricultural production (5–7), animals (8,9), and humans (10–12).

A complication faced by MEGARes and other ARG sequence repositories is the inclusion of ARGs whose AMR properties are only conferred when specific single nucleotide polymorphisms (SNPs) and insertions and deletions (indels) are contained within the gene sequence (2,13). These specific loci confer resistance through effects on gene expression via mRNA transcription, protein conformation, and mRNA/protein localization (13–16). While this issue impacts a minority of ARGs within the total ARG database (i.e. 589 out of 8,733 accessions in MEGARes v3.0), some ARG classes are heavily impacted by this special circumstance. For example, nearly 40% of the MEGARes v3.0 ARG accessions within the fluoroquinolone class of antibiotics require specific genetic variants to be considered resistance genes.

In MEGARes v2.0, we highlighted this issue for users by adding the flag ‘RequiresSNPConfirmation’ to the relevant ARG accession headers. In addition, we extended AMR++ pipeline to create AMR++ v2.0, which provides an option for users to integrate software tools developed by other research groups to confirm the presence of required SNPs (17,18). However, the integration of these tools with AMR++ v2.0 is often challenging given the nature of updates and maintenance work across many different software tools and research groups. Furthermore, searching the existing literature for information regarding the specific loci that confer resistance properties is a very time-intensive task, and this information has been sporadically available and updated within existing resources. Therefore, in this update to MEGARes and AMR++, we have significantly expanded the handling of these ARGs requiring variant confirmation by both improved annotation of loci of resistance-conferring genetic variants, as well as the inclusion of a novel algorithm for verification of these variants. While there are a variety of resources for verifying the presence of critical variants within individual sequences, prior to release of MEGARes and AMR++ v3.0, there were few efficient, high-throughput methods for confirming resistance-conferring variants (i.e. SNPs and other genetic variants) within unassembled, untranslated metagenomic data, which led many researchers to perform meticulous validation of individual sequence reads, which is highly tedious and time consuming (19). Creating a suitable high-throughput computational approach for automating this validation process was a high priority in developing updates for MEGARes and AMR++.

## UPDATES TO MEGARes AND AMR++

### Updated ARG accessions for MEGARes v3.0

As described (1,2), previous versions of MEGARes included 7868 unique, functionally confirmed ARG sequences that were compiled from multiple public genomic repositories including ResFinder (20), ARG-ANNOT (21), the National Center for Biotechnology Information (NCBI) Lahey Clinic beta-lactamase archive, the Comprehensive Antibiotic Resistance Database (CARD) (18), NCBI’s Bacterial Antimicrobial Resistance Reference Gene Database (22) and BacMet (23).

To identify a list of accessions to evaluate for inclusion in MEGARes v3.0, all sources of ARG sequences that had been used in creation of previous MEGARes versions were compiled and compared to MEGARes v2.0. Several of the original sources are no longer updated, and so updates for MEGARes v3.0 were derived from the most current versions of the CARD, NCBI’s Bacterial Antimicrobial Resistance Reference Gene Database, and ResFinder. The CARD database (downloaded September 2022) is organized in five gene groups: protein homolog (*n* = 4634), knockout (*n* = 19), over-expression (*n* = 13), variant (*n* = 171) and rRNA gene variant (*n* = 84) models. As confirming the absence of a gene is arguably impossible with a metagenomic sequencing approach due to the risk of false negatives, gene accessions from the knockout model were excluded for this update. NCBI’s Bacterial Antimicrobial Resistance Reference Gene Database, which contained 1568 sequences, was obtained from its distribution with the AMRFinderPlus software (downloaded July 2022). Finally, ARG accessions in the ResFinder database (*n* = 3154), which emphasizes acquired resistance, were also included in the update (downloaded 2022/07/28). ResFinder was recently expanded to include the tool PointFinder (17), which detects chromosomal point mutations that mediate resistance in a select group of bacterial species. Therefore, PointFinder accessions were considered for inclusion in MEGARes v3.0.

The ARG sequences downloaded from each source database were clustered with MEGARes v2.0 gene accessions by 100% sequence similarity to remove redundant accessions using CD-HIT (version 4.8.1.) (24). For all source databases, a single representative sequence for each cluster was saved and all novel sequences not clustered with MEGARes v2.0 sequences were concatenated into a single file (*n* = 1072). As MEGARes focuses on bacterial resistance to antimicrobial compounds, gene accessions from NCBI’s Bacterial Antimicrobial Resistance Reference Gene Database that were associated with ‘Heat’, ‘Virulence’, or anti-malarial resistance were removed from the update. Novel genes from each source were combined into a single file and they were checked for redundancy using CD-HIT-EST with the following parameters: -G 0 -c 1.0 -AS 0 -AL 0 -AI 1.0 -aS 1.0. This resulted in a total of 865 new ARG accessions, representing 89 gene groups, 13 resistance mechanisms and 2 new drug classes being added in MEGARes 3.0, for a total of *n* = 8733 ARG accessions.

We then hand-curated the new ARG accessions to incorporate them into the MEGARes hierarchical acyclic classification scheme, as previously described (1,2). Briefly, we employed CD-HIT to cluster gene accessions using 80% sequence similarity and referring to both the source and existing MEGARes annotations to determine the appropriate classification. The headers for gene accessions requiring the presence of SNPs to confer resistance were additionally modified to include ‘RequiresSNPConfirmation’. This label is unique to MEGARes and facilitates the processing of these sequences for further *in silico* testing and confirmation (described below). In addition, annotations were partitioned into two different annotation files to support various resistome analysis scenarios: the file ‘megares_annotations_v3.0.csv’ contains annotations for all types of antimicrobial compounds (i.e. drugs, biocides and metals), while the annotation file ‘megares_drug_annotations_v3.0.csv’ consists of annotations only for accessions that confer resistance to antimicrobial drugs. MEGARes is also distributed with the file ‘megares_to_external_header_mappings_v3.00.csv’ which includes the original headers for each accession (i.e. the header from the original source database); the MEGARes header and any relevant notes regarding the accession's annotation.

### Added resistance-conferring variant information to MEGARes 3.0 annotations

The MEGARes v2.0 (2) database contains 490 ARG accessions that require presence of specific variants in order to be considered resistance-conferring gene variants. Here, we confirmed the loci of these specific resistance-conferring variants within sequence reads, which were obtained from previously compiled sources, and supplemented with a manual search of the primary literature.

#### Extraction of variant loci from Meta-MARC and CARD

Meta-MARC uses DNA-based Hidden Markov Models to probabilistically classify ARGs into three different groups, of which the Group II model consisted of gene sequences requiring variant confirmation (25). The code and resulting models include a variant search file for Group II, which includes the loci of the resistance-conferring variants for each relevant Group II ARG accession (see https://raw.githubusercontent.com/lakinsm/meta-marc /master/src/mmarc_snpsearch_metadata2.txt). To use this information, the Group II gene sequences in Meta-MARC were mapped to the MEGARes v2.0, allowing us to identify the variant information for 138 ARGs. Every genetic variant identified within the Meta-MARC documentation was a single missense variant, i.e. only one SNP was needed for the gene to be considered resistant, and none of the variants induced a sudden stop in transcription of the sequence. A single error was identified in the Meta-MARC variant data: one TUFAB gene was <300 amino acids in length and therefore, could not contain the requisite resistance-conferring SNP at position 316. This error is logged in GitHub (https://github.com/Isabella136/AmrPlusPlus_SNP/blob/in-depth/genes-issues.md).

Additional resistance-conferring variants were recovered from CARD (18) using KARGVA, a program adapted from KARGA (26,27). Both KARGA and KARGVA perform *k*-mer-based ARG analysis, with KARGVA being specifically for the identification of resistance conferred by variants. The code for KARGVA, made public on GitHub, includes a database containing both variant information and the matching MEGARes v2.0 headers (see https://raw.githubusercontent.com/DataIntellSystLab/KARGVA/main/kargva_db_v5.fasta). Combined with the information extracted from Meta-MARC, the KARGVA information expanded the set of ARGs with known resistance-conferring loci from 138 to 267. In contrast to Meta-MARC, CARD’s ARG information also includes resistance-conferring insertions and deletions (indels), single resistance-conferring nonsense or nonstop variants, resistance-conferring frameshifts, and n-tuple resistance-conferring variants. For example, one entry included a suppressible frameshift (28). CARD sources this information from the primary literature, i.e. from one or multiple original source papers. For each curation from CARD, we identified the loci of the variant in nucleotide sequence and the amino acid sequence to identify any errors. This curation revealed several errors, most of which were caused by copying the wrong position or amino acid from the original source paper to CARD, or from CARD to KARGVA; or by mislabelling a previously discovered susceptible or neutral variant as a resistant variant. Other errors arose from the fact that the ARG sequence used in the original source paper was not the same as the ARG sequence used in CARD, or that the sequence in CARD was not the same as the one in MEGARes; such differences led to errors in the position of the relevant variant, since the ARG sequences were slightly different between the various resources. Finally, due to the nature of how CARD retrieves genetic variants from the literature, some variants were often referenced multiple times. All issues from KARGVA, CARD, and Meta-MARC were compiled into one file, published on GitHub along with the program used to confirm the variants (see https://github.com/Isabella136/AmrPlusPlus_SNP/blob/main/genes-issues.md). Two ARGs from CARD were omitted as they could not be aligned with the corresponding MEGARes sequences, and four additional CARD accessions were omitted because the relevant polymorphisms did not reduce phenotypic susceptibility (i.e. they were not resistance-conferring variants).

After these corrections and omissions, our list of ARGs with complete and accurate variant information comprised 261 unique ARGs within the MEGARes v2.0 accessions. All *Mycobacterium tuberculosis*-specific ARGs that require specific polymorphism sequences for function, as well as an overwhelming majority of ‘RequiresSNPConfirmation’ ARGs that confer resistance to elfamycins, fluoroquinolones, fosfomycins, lipopeptides, and rifampin are included in these 261 ARGs. However, the use of information from Meta-MARC, CARD and KARGVA did not include information on any of the ‘RequiresSNPConfirmation’ ARGs within the classes that cover macrolide-lincosamide-streptograminB (MLS) or phenicol antibiotic compounds; and very few such ARGs within the tetracycline and trimethoprim antibiotic classes. Lastly, a majority of ribosomal subunit genes remained unaccounted for by the use of these resources.

#### Addition of new variant location information through primary literature search

To expand the variant information for ARGs not contained within Meta-MARC, CARD or KARGVA, we performed a comprehensive literature search. To begin the literature search process, we used information contained within the ARG header to search for the microbial organism and gene, in conjunction with the antibiotic to which the organism and gene are known to confer resistance. For example, a search on PubMed with the search string ‘Clostridium difficile GYRA resistance to fluoroquinolones’ yielded valuable results, with primary literature containing phenotypically verified variant information for relevant ARGs. However, the majority of ARGs that required resistance-conferring variants (∼68%) did not have a microbial organism contained in the MEGARes v2.0 header. In these cases, the DNA sequence of the ARG (obtained from the MEGARes v2.0 database) was entered into the NCBI Basic Local Alignment Search Tool (BLAST) using both the BLASTN and the translated nucleotide tool BLASTX search options (29). Results were used to determine what organism(s) were associated with each ARG containing a resistance-conferring variant; and these organisms were then used to search the literature, as described above.

The literature available was highly dependent on the ARG group being investigated. Nonetheless, variant information was added for 76 ARGs through this method. ARGs from classes such as MLS, phenicols, sulfonamides, rifampin, trimethoprim, tetracycline and aminocoumarins sometimes lacked variant information for the identified organisms and genes, indicating that variants within these ARGs have not yet been researched extensively. Such genes included catB, DHFR, folP, ompF, parE, rpoB and tetR. However, our search process yielded some primary source literature with relevant variant information for all investigated ARGs and their associated organisms, even though this information was occasionally very sparse. The loci for resistance-conferring variants for ARGs from other classes have been researched more extensively, and therefore the body of literature contained copious amounts of information on resistance-conferring variants across many bacterial organisms. A few of the well-researched ARGs had associated literature published in the 1980s or 1990s, in addition to recent publications. The more current pieces of literature often noted several additional variants beyond those reported in older publications.

#### Development of a new database and annotation scheme for resistance-conferring variants

To make the retrieval of variant information easier, a new database of resistance-conferring variants was introduced as an addition to the already established MEGARes database. The annotation scheme used for ARGs remained the same; however, replacing the ‘RequiresSNPConfirmation’ label is a list of resistance-conferring variants and indels as well as a ‘context’ of five amino acids on either side of each variant. This context was already provided by Meta-MARC (25) in the variant search file. We note that because variants within Meta-MARC are determined by model type, sometimes the context can differ between genes from the same model by one amino acid. Therefore, to denote this difference, instead of one amino acid, the context would instead contain a list, denoted by brackets, of amino acids that can be found at this position. This annotation scheme was kept in the updated MEGARes database.

Retrieving context from CARD (18) was more complicated; for each ARG, the CARD sequence had to be recovered, and from there the five amino acids to the left and to the right of each variant were isolated. As previously mentioned, sometimes the sequence provided in CARD would not match with the location of the resistance-conferring variant, and therefore, the reference sequence used in the papers listed in CARD had to be recovered instead. Other times, the sequence in CARD was different from the sequence in MEGARes, which we addressed by alignment of the two sequences, using Biopython (30), in order to find the true mutation positions as well as the true context. Moreover, *n*-tuple variants listed in CARD that contain at least one single mutation that can confer resistance by itself were removed in this database to reduce the complexity of the variant verification process; this impacted 11 ARGs in CARD, encompassing 56 out of 70 *n*-tuple variants across these 11 ARGs.

The variants retrieved from the literature were individually mapped to the relevant sequence in MEGARes. Many of the variants–especially for the rRNA subunit sequences–were based on loci in the *Escherichia coli* genome. Therefore, pairwise alignments were performed using either BLAST (29) or EMBOSS (31) between the MEGARes sequences and the *E. coli* reference genome to determine the loci of the variant. Once this process was done, the MEGARes sequence was used to retrieve the context. For variants in the rRNA subunit sequences, since those sequences do not get translated, the context consisted of the five nucleic acids on either side of the variant.

In addition, a new annotation scheme was created to convey information on each variant listed. To differentiate between each type of variant, a short-hand notation was developed. ‘Mis’, ‘Del’, ‘Ins’, ‘Nonsense’ and ‘Nonstop’ represent a missense variant, a deletion, an insertion, a nonsense variant and a nonstop variant, respectively, while ‘Nuc’ and ‘NucDel’ represent a nucleic acid missense variant and a nucleic acid deletion, respectively. ‘Mult’ or ‘NucMult’ represent an n-tuple amino acid or nucleic acid variant respectively, and each variant in an *n*-tuple variant also has one of the five notations mentioned previously. ‘Hyper’ represents a variant that causes a gene to be hypersusceptible to an antimicrobial. ‘Must’ represents a group of amino acids/nucleic acids required for intrinsic resistance to antimicrobials (which we refer to as a *must* group); additionally, the inclusion of ‘Amino’ or ‘Nuc’ in front of each *must* group member indicates its nature as either an amino acid or a nucleic acid.

For ARGs that can also have resistance-conferring frameshifts, an additional label was added; this label precedes the list of resistance-conferring SNPs and indels described above. This additional label contains the prefix ‘FS’ and is followed by the mechanism by which the corresponding ARG can sustain a frameshift without endangering the organism. For example, many of the relevant ‘FS’ ARGs confer resistance through a change in their involvement in target synthesis or pump repression; drug activation; 16S methyltransferase activity; and porin formation or transport. However, several of these frameshift ARG variants do not have a clear underlying mechanism and are therefore labeled as ‘miscellaneous’. Additionally, one gene has been shown to allow for a specific frameshift in its N-terminal, creating a stop codon (with potentially another start codon later in its sequence) (32). Although this frameshift has yet to be proven as resistance-conferring, it has still been given an FS label.

While there is a common annotation scheme for missense variants (i.e. the wild-type amino acid/nucleic acid, followed by the position, followed by the mutant amino acid/nucleic acid), this doesn’t seem to be the case for nonsense variants and deletions. Most nonsense variants annotations in CARD replace the mutant amino acid with a ‘STOP’; however, instances of an ‘X’ replacing the mutant amino acid have also been found, indicating that multiple nonsense variant annotation schemes exist. To avoid confusion, we implemented a single annotation scheme for all nonsense variants, which uses an asterisk to represent a stop codon. This approach was favored to avoid confusing the ‘X’ as an unknown amino acid or the ‘STOP’ as a list of mutant amino acids.

Deletions also have numerous annotation schemes in CARD. While CARD invariably uses a dash (‘−’) at the beginning of the annotation to represent deletions, this dash is variably followed by either the wild-type amino acid/nucleic acid, or its position. Due to our use of the ‘Del’ short-hand notation at the beginning of our MEGARes annotations, neither of these two annotation schemes were necessary; instead, only the wild-type amino acid/nucleic acid and the position are listed, in that respective order.

Conversely, insertions only have one common annotation scheme in CARD: a plus (‘+’) sign to indicate insertions, followed by the inserted amino acid(s) and its/their position. With the ‘Ins’ short-hand notation the plus sign is not necessary in MEGARes v3.0; instead, only the inserted amino acid(s) and position are listed. Likewise, even though a common annotation scheme for nonstop variants exists in CARD (i.e. the three-letter notation ‘Ter’, which stands for ‘termination’, followed by the position and the mutant amino acid or the two-letter notation ‘fs’, respectively), we have replaced this with the ‘Nonstop’ notation in relevant MEGARes v3.0 headers. Although the annotation scheme for missense variants is consistent across all relevant CARD entries, we modified this scheme slightly to accommodate the resistant-conferring mutants in that position, thus facilitating verification of the variants. Finally, ARGs that have the previously defined *must* groups only have the required amino acids/nucleic acids and positions in their annotations.

### Resistance-conferring variant identification

Using the resistance-conferring variant database that we described in the previous section; we developed a method that identifies whether these specific variants occur based on the read alignment. We briefly describe this identification here and leave a detailed description to the supplement. We remind the reader that one of the initial steps in the AMR++ pipeline is to align the sequence reads to the MEGARes database using BWA, which produces a Sequence Alignment Map (SAM) file (33). This SAM file has a line for each alignment produced by BWA, and each line has at least 11 fields that are tab delimited. The fields that we use include: (i) the identifier of the read, (ii) the ARG of the alignment and (iii) the CIGAR string that describes the nucleotide-pairing of the alignment. This SAM file and the database of resistance-conferring variants is the only information that is needed for this verification step.

Given a specific alignment in the SAM file, the first step is to identify whether it coincides with an alignment to an ARG gene that requires confirmation of a resistance-conferring variant and if so, what type of ARG it is. To facilitate the identification of variants, we have categorized all ARGs requiring variant confirmation into five different categories of gene types based on the type of variants they can contain.

- An I-type gene corresponds to a gene that is intrinsically resistant to an antimicrobial.
- S-type, F-type, or H-type genes correspond to genes that have a suppressible base pair insertion, a non-suppressible frameshift, or a hypersusceptible variant, respectively.
- Lastly, an N-type gene corresponds to a gene that belongs to neither of the previous categories.

**Figure 1.**
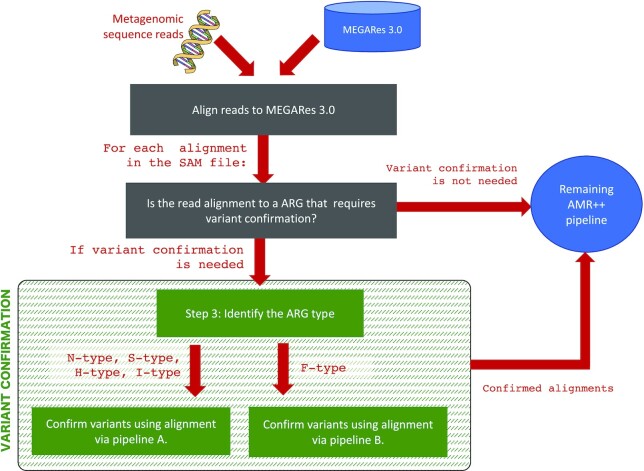
An overview of AMR++ with resistance-conferring variants confirmation. After running the variant confirmation pipeline for each alignment to an N-type, S-type, H-type, I-type or F-type ARG, the remaining part of the AMR++ pipeline is run on the confirmed read alignments, and the read alignments that do not need confirmation. Pipeline A and pipeline B are shown in the Supplement.

The current MEGARes v3.0 database contains only one S-type gene and one H-type gene. Regarding the previously mentioned gene that allows for a non-resistance-conferring, nonsense-causing frameshift, it is also considered as an N-type gene and will be subsequently referred to as the N-type ‘FS’ gene. Each ARG requiring a resistance-conferring variant is categorized into one of these types. Next, depending on the ARG type, the verification of the variant is led into two pipelines. If the ARG, is I-type, S-type, N-type or H-type then one pipeline is used (shown in Figure 1 in the Supplement) and otherwise a slightly modified pipeline is used (shown in Figure 2 in the Supplement). The pipelines differ on how frameshifts and the amino acid alignments are considered. We note that the amino acid alignment can also be inferred by the information in the SAM file. Each pipeline leads to either confirmation that the resistance-conferring variant exists for that alignment; or does not. This is completed for each alignment in the SAM file. The aggregate information is then used by the remaining part of the AMR++ pipeline to verify the presence or absence of each ARG, and an updated count matrix for all ARGs in MEGARes v3.0 is outputted to the user. An overview of the AMR++ pipeline with variant confirmation is shown in Figure 1.

### Updated AMR++ 3.0: NextFlow and SnakeMake pipelines

The AMR++ bioinformatic pipeline was designed to interface with MEGARes and facilitate resistome analysis using short-read sequencing data. Given a set of metagenomic sequencing samples, AMR++ can be used to perform quality trimming, host sequence removal, and alignment of all samples to the latest MEGARes database. AMR++ produces a count matrix with the number of alignments to AMR gene accessions for each sample. In response to feedback from AMR++ users, the updates for version 3.0 are tailored to facilitate common use cases, address reported computing challenges, and incorporate the variant confirmation software described above.

The nextflow scripting language (34) was used to write AMR++ and this was transitioned to use of the DSL2 syntax extension for this 3.0 update, allowing for the pipeline to be organized into modules that can be strung together into workflows. By making this extension, AMR++ 3.0 now provides the user with increased control over the steps undertaken in the analysis pipeline. For example, users can skip the time-intensive step of host removal for samples with high background host DNA. Further, users could choose to perform the pre-processing steps using other bioinformatic software, and only employ AMR++ for resistome analysis including alignment, rarefaction analysis with plotting, integration with software for variant confirmation, and creation of count matrices.

With a commitment to promoting resistome analysis in both proprietary and non-proprietary computing clusters, AMR++ was designed to facilitate use based on your computing resources. It is typical to face permission issues in large computing clusters, with users often being limited in their ability to install new software. In version 3.0, AMR++ provides the option to manage all software dependencies using anaconda, allowing for easy installation of all necessary bioinformatic tools without the need for ‘sudo’ permissions or requesting software installation. AMR++ 2.0 was implemented with a Singularity container hosted on Singularity-hub, but due to reduced support for continued container image hosting, all dependencies are now included in Docker containers hosted on docker-hub (35). Fortunately, these docker containers can also be seamlessly employed using the Singularity software (36).

Another major concern when running bioinformatic analysis pipelines is storage requirements. Using nextflow to write the AMR++ pipeline has numerous benefits, including the ability to use the ‘-resume’ flag to restart a failed pipeline run. However, this functionality requires the use of temporary files that can double or triple the total amount of storage space required to complete the entire pipeline. Using the flag ‘–pipeline’, AMR++ can now choose which sections of the pipeline to run and break up the storage requirements into manageable quantities. Alternatively, we present AMR++ written in Snakemake (37), a python-based workflow engine. This pipeline is optimized to reduce storage requirements and dependencies while replicating the steps of the entire AMR++ workflow.

Extended documentation is available on the MEGARes website (http://meglab.org) and the AMR++ github repository (https://github.com/Microbial-Ecology-Group/AMRplusplus). The steps of the AMR++ pipeline have been previously described in detail (1,2). These analytic steps have largely remained unchanged and significant updates to the pipeline are described below.

#### Quality assessment, trimming, and host removal

When running the standard AMR++ pipeline (–pipeline standard_AMR), raw sequencing reads are first assessed for quality using the fastQC software (38) and reports from all samples are aggregated into an easy to read report created using multiQC (39). Then, using Trimmomatic (40), reads are filtered for quality and to remove sequencing adapters. To identify host sequences from a user-provided genome, quality trimmed reads are aligned to the presumptive host genome using Burrows-Wheeler-Aligner (BWA) (33). Then, BEDTools (41) is used to remove all host-associated reads. Running the truncated pipeline with the fast AMR++ pipeline (–pipeline fast_AMR) will perform the quality trimming and skip the host-removal step to reduce running time and storage requirements. A custom python script is then used to provide filtering statistics in tab-delimited text files for all samples.

#### Resistome analysis

As part of the AMR++ pipeline, the high-quality, non-host reads are then aligned to the MEGARes database using BWA-MEM (33). The resulting SAM alignment file is then used for (a) resistome characterization, and (b) rarefaction analysis with plotting of all samples. Resistome analysis consists of using ResistomeAnalyzer, a custom C++ program that applies the user-defined criteria, gene fraction, to reduce false-positive classification. Gene fraction is defined as the minimum proportion of nucleotides in a reference sequence that must have at least one read aligned to it for the gene to be considered ‘present’ in a sample. By default, AMR++ applies an 80% gene fraction threshold with ResistomeAnalyzer and the filtered data are summarized as a count of aligned reads for each gene accession in each sample. Starting at the gene accession ID level, counts are aggregated upward in the hierarchical classification structure to the group, mechanism, class, and resistance type levels. Using a custom python script, a count matrix is produced summarizing the counts at the gene accession level for all samples in the file ‘AMR_analytic_matrix.csv’. Concurrently, AMR++ utilizes Samtools (42) to filter out duplicate sequences and provide a method for assessing PCR duplication. De-duplicated results are similarly reported in the AMR++ pipeline output in the file ‘AMR_deduped_analytic_matrix.csv’.

AMR++ 3.0 then incorporates the variant confirmation software described above by analyzing each SAM alignment line to identify variant-confirmed alignments. In addition to the count matrix of alignments to all gene accessions, AMR++ also reports a modified count matrix in the file ‘AMR_analytic_matrix_with_SNP_Verification.csv’. For the ARGs requiring variant confirmation, 80% gene fraction is required in addition to the requirement of resistance-conferring variants. Finally, the SAM alignment files are evaluated using RarefactionAnalyzer, a custom C++ program that performs rarefaction analysis to assess sequencing depth. Results are reported at the type, class, mechanism, group, and gene level which were used with a custom python script to plot results for all samples at each taxonomic level.

#### Microbiome characterization

The ability to also perform microbiome analysis with AMR++ 3.0 is available in addition to the standard workflow (–pipeline standard_AMR_wKraken). The k-mer based metagenomic classifier Kraken2 (43) is used to analyze the high-quality, non-host reads. Kraken2 is run with default parameters and with the most conservative scoring, using the highest value for the ‘-confidence’ flag (i.e. 1). Results are summarized using a custom python script that reports counts starting at the species level, with strain-level counts aggregated to the species level.

## DISCUSSION

In this paper, we release versions 3.0 of both MEGARes and AMR++ (downloads and documentation available at https://www.meglab.org). The most significant advancement in this release was the addition of a variant verification method that enables resistance-confirming variants to be identified from non-assembled, non-translated metagenomic sequence reads. The integration of this tool with the MEGARes ontology and updated AMR++ pipeline enables users to generate a combined count matrix, encompassing ARGs that do and do not require specific variants for their resistance function. Importantly, this count matrix can be generated from a single workflow. Previously, users of MEGARes had to handle the ‘RequiresSNPConfirmation’ ARGs separately from all other ARGs. In practice, many users decided to drop these ARGs from their metagenomic analyses to simplify the bioinformatic workflow. Alternatively, researchers would attempt to confirm the presence of specific variants using available software such as RGI (18) or PointFinder (17). However, these tools required high-quality assembled and/or translated data, which are difficult or even impossible to generate for metagenomic datasets (44). From this perspective, MEGARes and AMR++ v3.0 represent important usability advances for the metagenomic research community. Indeed, for some ARG classes, this new variant verification functionality substantially expands the number of ARGs that can be included in a resistome analysis. Our intensive primary literature search also expanded this capacity by compiling the loci for variants in 337 ARGs; this intensive work of compiling loci information will continue, with the goal of covering all relevant ARGs. To support use of this new tool by the research community, we have made all underlying data and scripts publicly available; and we have expanded the usability of AMR++ 3.0 by adding modulizable NextFlow workflows and a SnakeMake distribution.

## DATA AVAILABILITY

The MEGARes 3.0 reference database and annotation files are available at https://www.meglab.org. All AMR++ NextFlow pipeline source files are freely available on GitHub at https://github.com/Microbial-Ecology-Group/AMRplusplus. The SnakeMake AMR++ pipeline version files are available under the MIT license on https://github.com/jonathan-bravo/amrplusplus_v2. The variant verification program and further details on the SNPs and Indels are available under the GPL3 license at https://github.com/Isabella136/AmrPlusPlus_SNP.

## ENDNOTES

Anaconda Software Distribution. (2020). Anaconda Documentation. Anaconda Inc. Retrieved from https://docs.anaconda.com/.

## Supplementary Material

gkac1047_Supplemental_File
