# Supplementary material for: MEGARes and AMR++, v3.0: an updated comprehensive database of antimicrobial resistance determinants and an improved software pipeline for classification using high-throughput sequencing
Source: Nucleic Acids Res. 2022 Nov 16;51(D1):D744–52. doi: 10.1093/nar/gkac1047 (PMC9825433; doi:10.1093/nar/gkac1047)
Supplement: gkac1047_Supplemental_File [file gkac1047_supplemental_file.docx]

**SUPPLEMENTARY INFORMATION**

***Variant verification method***

**Step 0: Short Read Alignment.** We remind the reader that one of the first steps of the AMR++ pipeline is to take the short reads and MEGARes database as input and align the reads using BWA to MEGARes database. This alignment step produces a Sequence Alignment Map (SAM) file that describes each individual alignment. If a read aligns X times to the MEGARes database, then there will be X lines in the SAM file that describes each alignment. Each line is tab delimited with at least 11 different fields. We do not use each field but consider the fields that describe: (1) the ID of the aligned read; (b) the ARG that the read aligned to; and (c) CIGAR string that describes what each nucleotide in the read aligned to, including insertions and deletions. Hence, the SNP verification program considers each alignment line in the SAM and considers the following steps.

**Step 1: ARG Requirement.** Before starting the actual verification process, a requirement check is performed to identify whether the current alignment is to one of the 589 MEGARes reference sequences that require SNP confirmation. If the check is unsuccessful, i.e., the reference sequence aligned to the read does not require variant confirmation, then the program immediately moves on to the next alignment line in the SAM file. Otherwise, the program moves on to the first step of the confirmation process, which is to check for frameshifts.

**Step 2: Checking for frameshift variant.** It is first determined whether there exists a frameshift variant by considering the CIGAR string and calculating the difference between the total number of inserted nucleotides and the total number of deleted nucleotides in the query sequence. We note that rRNA subunit sequences cannot have frameshifts so in fact we omit this check if the ARG is a rRNA subunit sequence. The presence or absence of a frameshift variant has different outcomes based on the ARG type. There exist some edge cases (i.e., one or two ARGs) that do not follow this exact algorithm for which we check for individually. We do not go into the details of these cases but instead focus on the general algorithm.

- For I-type, H-type, and N-type ARGs, if there exists a frameshift then no conclusion can be made regarding whether the read contains the necessary resistance-conferring variants and therefore, the program immediately moves on to the next alignment in the SAM file.
- The frameshift check for a S-type ARG is slightly different than for I-type, H-type, and N-type and requires analyzing the CIGAR string of the alignment. If there is a frameshift in the alignment that cannot be suppressed, then no conclusion can be made regarding the presence of a resistance-conferring variant and we consider the next alignment in the SAM file. If a suppressible frameshift is found then the verification process continues until the nonsense variant check, during which the presence or absence of a nonsense variant would determine whether the read contains the necessary variant.
- For F-type genes, if there exists a frameshift then that alignment can be deemed as containing the requisite resistance variant.

**Step 3: Variant identification from nucleotide alignment.** Next, if we are in pipeline A (for I-type, H-type, N-type and S-type ARGs) then we determine whether the ARG in the alignment is an rRNA subunit sequence. If it is, then we consider the nucleotide alignment. Using the CIGAR string, each nucleotide in the reference sequence is mapped to its corresponding nucleotide in the sequence read. Considering this nucleotide mapping, we check for intrinsic resistance, which consists of verifying that all *must* group members are present. If the alignment is intrinsically resistant, then the alignment is confirmed to be resistant and the next alignment in the SAM file is considered. Otherwise, we cannot make any conclusion and continue with the next step in the pipeline.

For S-type ARGs, if a suppressible base pair insertion is found and is not followed by either two base pair insertions or one base pair deletion, then the insertion is suppressed in the nucleotide alignment. Since the nucleotide that is skipped during translation to make the suppression possible has previously been found, the program allows for this same nucleotide to be skipped during nucleotide alignment. Similarly, for the N-type “FS” gene, if a non-resistance-conferring, nonsense-causing frameshift is found, then the nucleotide alignment starts right after the stop codon. Because we don’t know which methionine codon would function as a start codon (or whether another codon would take that role), the program makes a safe assumption that would avoid disregarding potential amino acids from the shorter gene

Finally, if the ARG is not a rRNA subunit sequence or we are in pipeline B (for F-type ARGs) then we do not consider the nucleotide alignment and directly consider the inferred amino acid alignment (i.e. Step 4).

**Step 4: Variant identification from amino acid alignment.** Next, we consider the amino acid alignment, which can be inferred from the SAM file via codon walking from the start loci of the alignment. The codons in the read that contain inserted or deleted nucleotides or are between inserted or deleted nucleotides are treated differently than the other codons. The exception to this scenario exists with the ARG is a S-type ARG when there exists a suppressed nucleotide. Otherwise, in the case of insertions, the first codon in the read alignment with an inserted element is mapped to the first “shifted” condon in the reference sequence of the ARG. The subsequent codon in the read is mapped to this same reference codon. No more query codons get mapped afterward until finally an insertion occurs that would make the codons in the reference sequence of the ARG unshifted. When this occurs, both the codon in the sequence read with this insertion and the previous codon in the sequence read get mapped to this first “unshifted” codon. In the case of a deletion, the first codon in the sequence read with a deleted nucleotide gets mapped to the last “non-shifted” codon in the reference and the first “shifted” codon in the reference. Then, no codon in the sequence read is mapped until finally a deletion occurs that would make the codon in the reference “unshift”. When that occurs, the codon in the sequence read with the deleted element gets mapped to both the first “unshifted” condon in the reference and the previous codon in the reference.

**Step 5: Confirmation of resistance-conferring variants.** Once the amino acid alignment is obtained, it is used to identify the following variants.

- Nonsense variants. This consists of checking the translated sequence sequence for stop codons. If no stop codons are found, then we move onto the next variant identification. If a stop codon is found and the gene is an F-type, the read is marked as resistant. Otherwise, if the gene is not an F-type, then the first stop codon identified goes through resistance confirmation, which consists of looking through the list of possible nonsense variants listed in MEGARes to see if one of them matches. If confirmation is successful, then the read is considered to contain the resistance-conferring variant. If confirmation is not successful then the read is considered to not contain the resistance-conferring variants–nonetheless, we move to the next alignment in both cases.
- Intrinsic resistance. This consists of considering each mapping in the query sequence and confirming that all *must* group members are present. If all group members are confirmed then the read is considered resistant; otherwise, the next variant type is considered. This is not considered in pipeline B.
- Missense, insertions and deletions variants. This consists of a simple iterative search through the alignment to identify any SNPs, inserted codons, or deleted codons.
- N-tuple variants. This consists of the same confirmation method as the missense, insertions, and deletions variant confirmation except that more than one SNP, insertion or deletion must be present. If either variant is missing, then the read is considered to not contain the necessary resistance-conferring variant(s). If this confirmation is unsuccessful, then the read is considered to belong to a non-resistance-conferring sequence and next alignment is considered.
- Finally, we note that there exists one exception for a H-type ARG: if the hypersusceptible variant is found then the read is not considered to be resistance-conferring regardless of whether any other variant is present.

**Supplementary Figure 1: Pipeline A.** Overview of steps taken by the variant confirmation program for N-type, S-type, H-type, and I-type ARGs. When the program reaches either a red box or the blue box, it goes to the next alignment in the SAM file.
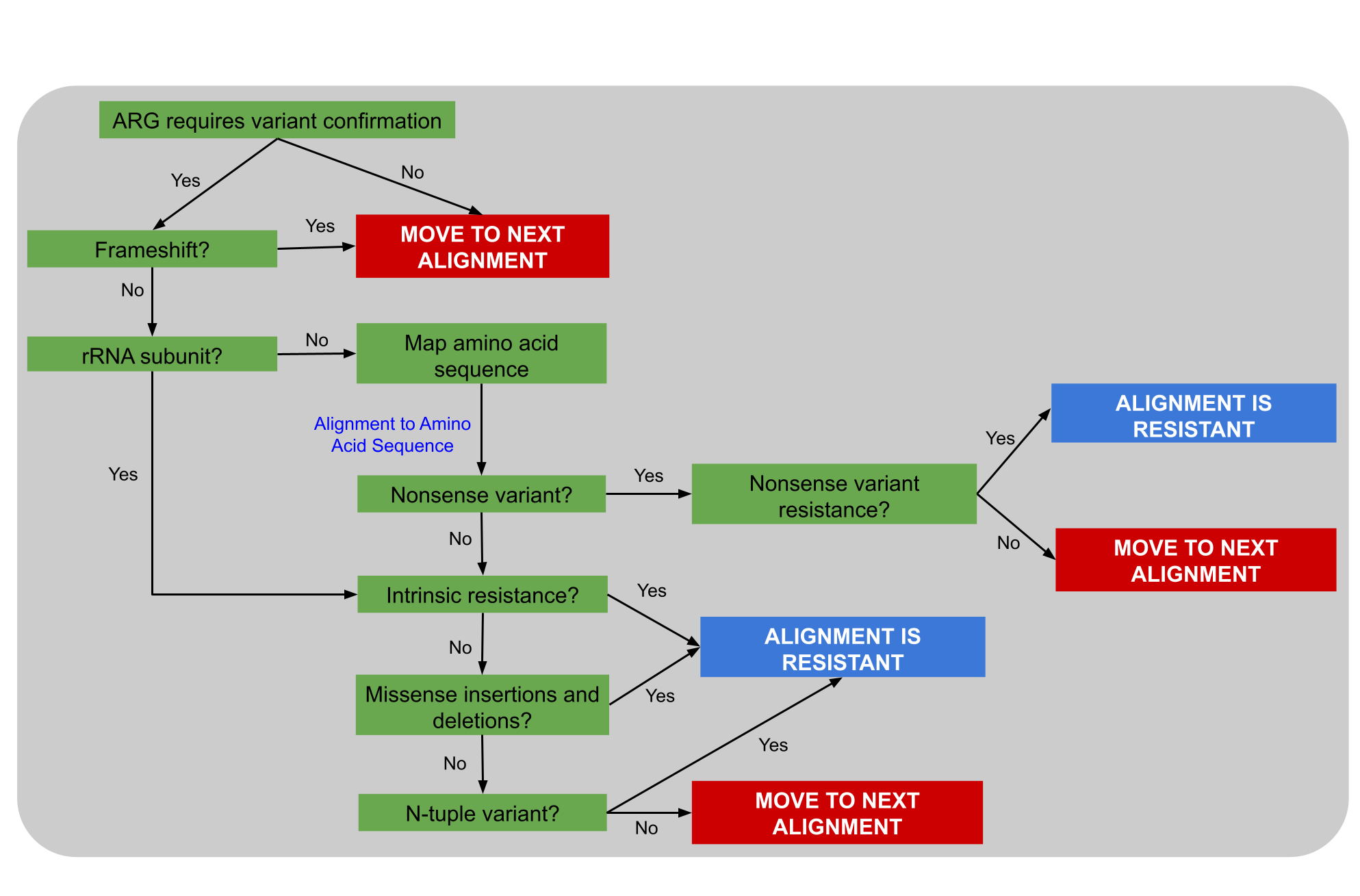


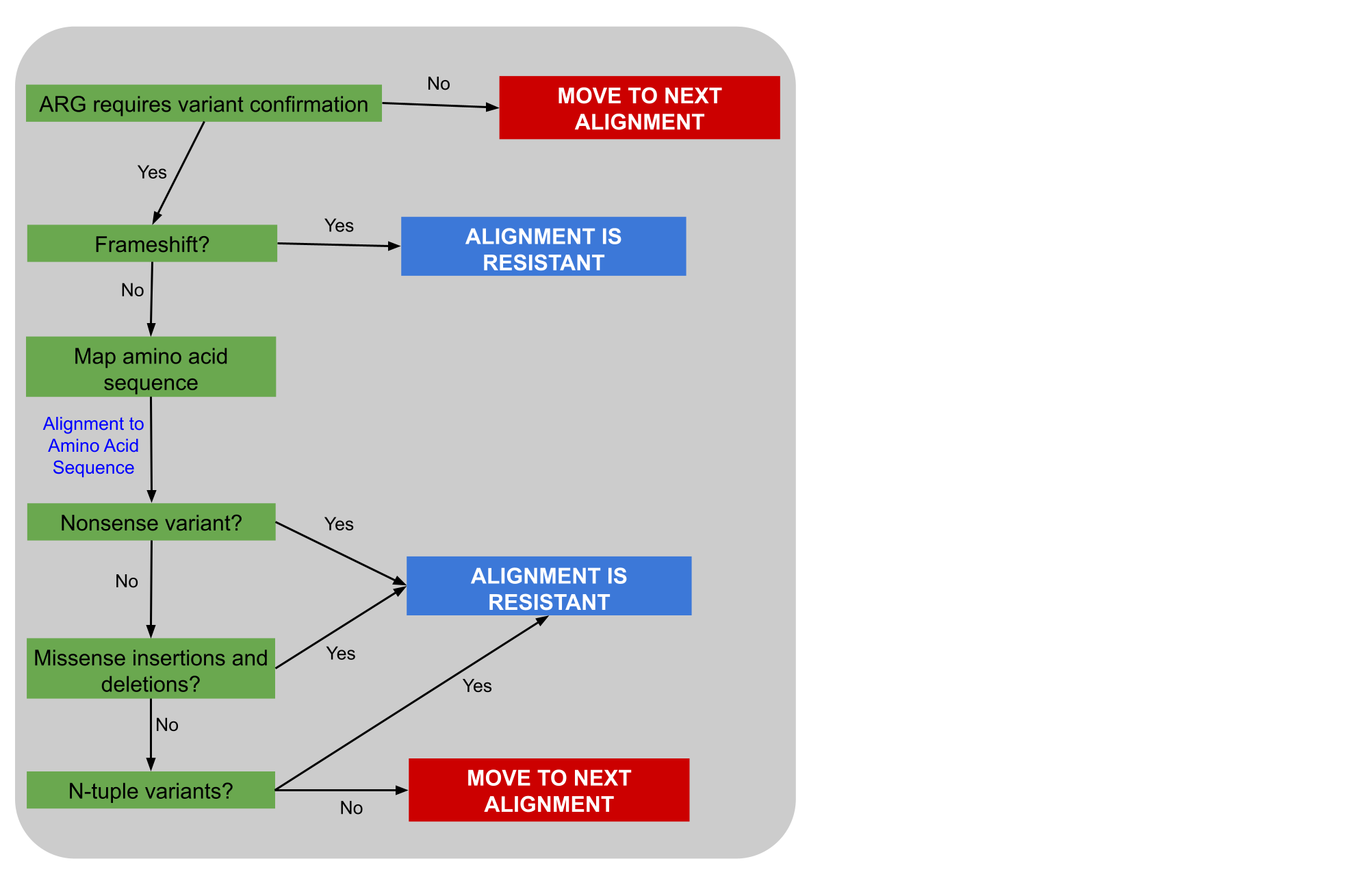


**Supplementary Figure 2: Pipeline B.** Overview of steps taken by the variant confirmation program for F-type ARGs. When the program reaches either a red box or the blue box, it goes to the next alignment in the SAM file
